# A Review on Current Trends of Polymers in Orthodontics: BPA-Free and Smart Materials

**DOI:** 10.3390/polym13091409

**Published:** 2021-04-27

**Authors:** Rozita Hassan, Muhammad Umar Aslam Khan, Abdul Manaf Abdullah, Saiful Izwan Abd Razak

**Affiliations:** 1 Orthodontic Unit, School of Dental Sciences, Universiti Sains Malaysia, Kelantan 16150, Malaysia; abdmanaf.abdullah@gmail.com; 2 BioInspired Device and Tissue Engineering Research Group, School of Biomedical Engineering and Health Sciences, Faculty of Engineering, Universiti Teknologi Malaysia, Skudai 81300, Malaysia; saifulizwan@utm.my; 3 Nanoscience and Technology Department (NS & TD), National Center for Physics, Islamabad 44000, Pakistan; 4 Med-X Research Institute, School of Biomedical Engineering, Shanghai Jiao Tong University, 1954 Huashan Road, Shanghai 200030, China; 5 Center for Advanced Composite Materials, Universiti Teknologi Malaysia, Skudai 81300, Malaysia

**Keywords:** adhesive materials, bisphenol-A, composite materials, dental resin, orthodontics, smart materials

## Abstract

Polymeric materials have always established an edge over other classes of materials due to their potential applications in various fields of biomedical engineering. Orthodontics is an emerging field in which polymers have attracted the enormous attention of researchers. In particular, thermoplastic materials have a great future utility in orthodontics, both as aligners and as retainer appliances. In recent years, the use of polycarbonate brackets and base monomers bisphenol A glycerolate dimethacrylate (bis-GMA) has been associated with the potential release of bisphenol A (BPA) in the oral environment. BPA is a toxic compound that acts as an endocrine disruptor that can affect human health. Therefore, there is a continuous search for non-BPA materials with satisfactory mechanical properties and an esthetic appearance as an alternative to polycarbonate brackets and conventional bis-GMA compounds. This study aims to review the recent developments of BPA-free monomers in the application of resin dental composites and adhesives. The most promising polymeric smart materials are also discussed for their relevance to future orthodontic applications.

## 1. Introduction

Polymer science has emerged as the most popular area of research due to its wide scope in modern materials engineering for improving structural and functional properties in clinical and biomedical applications. A wide range of polymers have optimum properties, and they further provide opportunities to enhance bioactivity, cytocompatibility, and antibacterial properties by chemical modification [1,2,3]. Various studies have indicated that polymeric composite materials may be potentially used to repair cartilage, bone, and heart valves, and may be employed as skin, hip, and dental implants in biomedical applications [4,5,6,7,8].

Orthodontics is a branch of science that deals with the diagnosis, prevention, and treatment of teeth-related problems including malpositioned teeth and jaws, and misaligned bite patterns. Dentofacial orthopedics is a branch of dentistry that focuses on modifying facial growth anomalies [9,10,11,12]. Misalignments of teeth and jaws are very common issues nowadays. According to the American Association of Orthodontics (AAO), approximately 50% of the population has malocclusions serious enough to benefit from orthodontic treatment. When implanting orthodontics that are medically necessary, this figure drops to less than 10%, according to the same AAO statement [13,14,15,16]. The health benefits and treatment of orthodontic lack strong scientific evidence. This is a serious issue in orthodontics that various researchers are trying to resolve using new materials and methods [17]. Treatment can last anywhere from a few months to a few years, and it entails the use of braces and other appliances to move the teeth and jaws around gradually. Jaw surgery may be performed in the case of severe malocclusion. Since the bones of children can move around more easily than those of adults, the treatment can be easier if it is started before, they reach adulthood to avoid complications [17,18,19,20].

Polymers have been extensively used as orthodontic materials due to their appropriate mechanical properties and esthetic quality. Orthodontic polymers could be classified into adhesives, brackets, and elastomeric modules and chains [21,22,23,24]. Various types of orthodontic polymers are commercially available, such as polycarbonate, polyurethane, polyethylene, polyamides, and polymethyl methacrylate, etc. [25,26,27]. Orthodontic materials should be biocompatible with the oral tissue, be nontoxic, and possess sufficient mechanical integrity in the oral environment during the entire treatment process [28]. A significant concern was raised in recent years regarding the release of Bisphenol-A (BPA), potentially derived from polycarbonate brackets or monomers, in the orthodontic composites bisphenol A-glycidyl methacrylate (bis-DMA) [29,30]. The elution of BPA may have arisen due to the incomplete polymerization of monomers present as an impurity after resin synthesis and polycarbonate degradation [31,32,33]. The negative effects of BPA include premature puberty and ovarian cancer in females and the disruption of the maturation of male reproductive organs, and increased levels of anxiety, depression, and problems in interpersonal relations in children [34,35,36]. Therefore, more productive research is required to overcome this issue by developing BPA-free orthodontic materials and improving the clinical procedures during the treatment to minimize BPA exposure. Another dental material trend involves developing smart materials that greatly enhance the conventional functions and properties of the materials. This paper will summarize the recent developments of non-BPA polymeric materials related to orthodontic treatments and will provide an overview of the concepts of smart materials and their future applications in orthodontics [37].

Martim et al. synthesized new multifunctional-urethane-methacrylate monomers for dental composites [38]. The conventional bis-GMA (bisphenol-A-diglycidylmethacrylate) and glycerol dimethacrylate (GDMA) were modified using nucleophilic addition reactions. The hydroxyl (–OH) groups, which are considered as good nucleophiles, react with highly reactive isocyanate (–NCO) groups of urethane-methacrylate precursors. These are electrophiles used to produce the new Urethane-(bis-GMA)-modified and Urethane-(GDMA)-modified monomeric systems [38,39]. The modifications were successful and confirmed by FTIR and ^1^H NMR. The physicochemical properties of the composites formulated with the newly synthesized monomers were evaluated. The degree of conversion (D.C. in %), volumetric shrinkage (VS in %), polymerization stress (P.S. in MPa), flexural strength (F.S. in MPa), flexural modulus (F.M. in GPa), water sorption (W_sp_ µg/mm^3^), and solubility (W_sl_ µg/mm^3^) were investigated [39,40,41,42]. Extraction studies in an organic solvent were also conducted to measure leachable components. Overall, the results showed that the degree of conversion for all composites prepared with U-(bis-GMA)-Mod and U-(GDMA)-Mod was lower than for those prepared with bis-GMA for diluent monomers triethylene glycol dimethacrylate (TEGDMA) and bisphenol A-glycidyl ethylacrylate (bis-EMA) by 6–9% on average, most probably due to the weaker hydrogen bond formation between N and H atoms in the urethane group, which presents a lower energy [43,44,45,46]. A comparison was also drawn between the newly synthesized monomers. U-(bis-GMA)-Mod displayed lower polymerization shrinkage and stress, lower water sorption and water solubility, and a higher value refractive index than U-(GDMA)-Mod [38,47]. However, U-(GDMA)-Mod had a greater resistance than U-(bis-GMA)-Mod to leaching in organic solvents, and both monomers were free of BPA. The authors concluded that U-(bis-GMA)-Mod was better than U-(GDMA)-Mod in terms of its higher refractive index, which would improve a match with commercial fillers due to the composite having better esthetic properties and due to a potentially higher depth of cure [48,49].

Furthermore, the lower water sorption and water solubility of U-(bis-GMA)-Mod is due to its greater hydrophobicity, making it ideal for use as an adhesive system that requires more hydrophobic monomers [50,51]. Theoretically, the hydrophobic bond system is expected to exhibit a better shear bond strength. However, a study revealed no significant differences in the shear bond strength of hydrophobic and hydrophilic systems after exposure to saliva [52,53]. A comprehensive and systematic study is needed to warrant the argument. Nevertheless, the composites derived from these two new urethane monomers have more advantages than those prepared with commercially available bis-GMA monomers, with a comparable polymerization shrinkage and stress, degree of conversion, improved mechanical properties, a faster rate of polymerization, and significantly lower water sorption and water solubility [54,55,56]. TEGDMA was the only leachate present from the composites prepared with U-(bis-GMA)-Mod and U-(GDMA)-Mod, whereas bis-GMA and TEGDMA were detected from composites prepared with bis-GMA [57]. The absence of BPA precursors during synthetic procedures is desirable in order to obtain non-BPA monomer systems.

**Figure 1:**
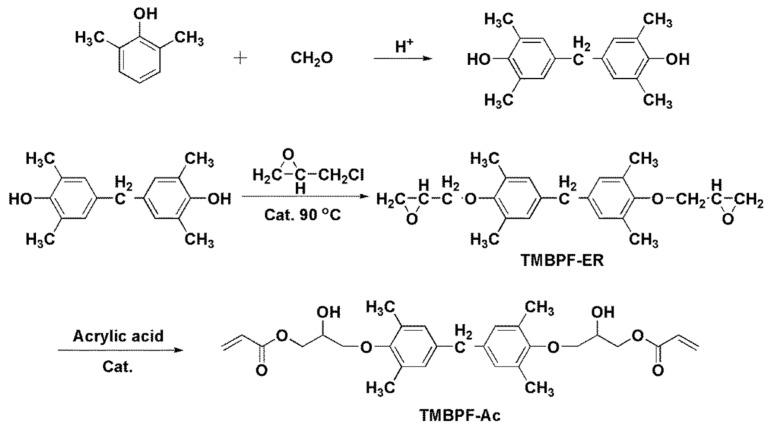
Illustration of the complete synthesis of TMBPF-Ac under the necessary conditions and reprinted with permission published work of Hong, L. et al. [60].

Nonetheless, the leachate of TEDGMA is undesirable as it may cause cytotoxicity to the surrounding host tissue. It should be noted that 4 mM of TEDGMA could reduce the human monocyte viability of cells by approximately 50% [58,59]. Therefore, the developed biocompatibility of the composite must be carefully investigated before a preclinical assessment. Hong et al. designed and synthesized a novel resin monomer (tetramethyl bisphenol F acrylate, human tetramethyl bisphenol F acrylate (TMBPF-Ac) using a multistep condensation reaction (Figure 1) [60]. First, tetramethyl bisphenol F epoxy resin (TMBPF-ER) monomer was synthesized as described in the previous work by Zhongguo et al. [61]. Then, TMBPF-ER was reacted with acrylic acid to produce TMBPF-Ac, and the results from the ^1^H NMR analysis showed that the synthesis of the monomer TMBPF-Ac was successful.

The characteristics and cytotoxicity of TMBPF-Ac-based resins relative to bis-GMA-based resins (bis-GMA/TEGDMA) were further investigated. TMBPF-Ac had a lower viscosity than bis-GMA. The double bond conversion (D.C.) values of TMBPF-Ac/TEGDMA and TMBPF-Ac were significantly higher than for bis-GMA/TEGDMA resin (*p* < 0.05) [60] as the structure of the TMBPF-Ac monomer had a smaller polarity and lesser interaction forces between molecules than those in bis-GMA/TEGDMA. The volume shrinkage (VS), water sorption (W_SP_), water solubility (W_SL_), and water contact angle (WCA) values were significantly lower in TMBPF-Ac resins compared to those in TMBPF-Ac/TEGDMA and bis-GMA/TEGDMA (*p* < 0.05) [60]. Evaluating these properties is important for dental materials as these values are related to cytotoxicity and biofilm formation. The highly cross-linked structures of TMBPF-Ac had inhibited water absorption [62]. Simultaneously, the higher surface energy of the TMBPF-Ac resin caused the molecules to become hydrophilic, preventing the adhesion of bacteria. It should be noted that microbes possess hydrophilic and hydrophobic surfaces whose adherence depends on their preference. Nevertheless, materials with a hydrophobic surface or a substrate with a low surface energy typically exhibit a reduction in biofilm formation as compared to their hydrophilic counterpart. The flexural strength (F.S.), flexural modulus (F.M.), indentation hardness (H_IT_), and indentation modulus (M_IT_) values of TMBPF-Ac/TEGDMA and TMBPF-Ac were also higher than those of the bis-GMA/TEGDMA resin. From all of the highlighted physical and biological properties, TMBPF-Ac-based resin is better than bis-GMA-TEGDMA resin. It has the potential to become an alternative to bis-GMA-based resin for dental composites in the future [63,64,65]. The basic material has been presented in the graphical abstract for the treatment of orthodontic.

## 2. Novel BPA-Free Composite Adhesives

**Figure 2:**
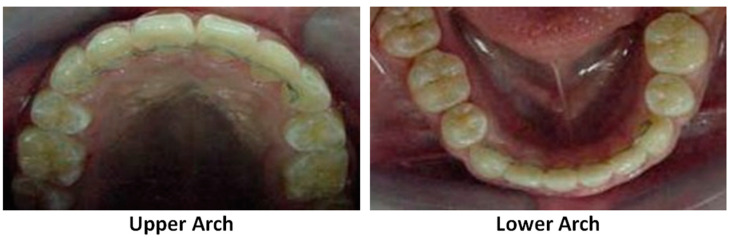
Illustration of the Lingual Fixed Retainer of the upper arch (**left**) and lower arch (**right**).

Iliadi et al. synthesized two experimental BPA-free resin composite adhesives for lingual fixed retainer bonding (Figure 2) [29]. The materials tested were: (1) EXA containing a single-aromatic ring highly reactive multifunctional monomer (PCDMA: phenyl carbamoyloxy-propane dimethacrylate), TEGDMA (triethyleneglycol dimethacrylate), UEDMA (aliphatic urethane dimethacrylate), and silanated glass (70 wt%) and catalysts; (2) EXB, which is based on aromatic-free urethane dimethacrylate monomers (TEGDMA, UEDMA, silanated glass (60 wt%), and catalysts); and (3) Transbond retainer bonding (TLR) adhesive containing bis-GMA, TEGDMA, silanated quartz (75–85 wt%), and catalysts that acted as a control. EXB presented the highest value of degree of conversion (DC) % among these three samples, followed by EXA and TLR. The lowest DC% in TLR may be due to the reduced conversion capacity of the bis-GMA monomer caused by the reduced segmental mobility of the C=C bonds attributed to the stiff bisphenol aromatic backbone, high molecular weight, and hydrogen bonding capacity of the monomer [66]. DC% was significantly improved in EXB as bis-GMA was substituted with the aliphatic UEDMA monomer. Thus, the steric hindrance phenomena associated with the bis-GMA structure were diminished. EXA represents a balance between the aromatic and aliphatic monomers as a mono-phenol dimethacrylate derivative was used to replace bis-GMA. The mechanical properties of the three samples were also assessed. TLR demonstrated the highest Martens Hardness (M.H.) and modulus of elasticity (E_IT_), followed by EXA and EXB. The reasons for the higher mechanical properties in TLR include the filler loading (TLR > 77 wt%, EXA 70 wt%, and EXB 60 wt%), the type of filler (TLR contains hard quartz, while both EXA and EXB consist of softer glasses), and the presence of bis-GMA in TLR. Bis-GMA can establish strong intermolecular hydrogen bonds through –OH groups and leads to greater mechanical properties. TLR was most affected for the water immersion test due to the hydrophilic ether bonds (–C–O–C–) of TEGDMA and the –OH groups of the bis-GMA molecules [67,68]. Pull-out strength testing did not show a statistical difference between the three samples tested, although TLR had higher mechanical properties. From these results, the authors proposed that the material containing the monoaromatic dimethacrylate (PCDMA) with greater hardness and elastic modulus values may be applied as an alternative to the bis-GMA-based adhesive [29,69,70]. This review summarizes the most significant attributes of polymers while also outlining the importance of BPA-free and smart materials. The recent researches about the design and development of BPA-free materials are summarized.

## 3. Clinical Recommendations to Minimize BPA Release

Despite altering the structure of monomers and molecular interactions, several clinical steps may also be considered to minimize the BPA release from orthodontic materials. It is recommended that one hold the light-curing tip of the lamp as close as possible to the composite. Applying indirect light-curing (around the edges of the bracket) instead of direct light-curing (through the bracket) reduces the exposure to BPA. Polishing the composite with a pumice stone after bonding could also reduce the potential for BPA. Finally, patients should wash their mouths during the first hour after the placement of the composite with a mouth-rinse or water to limit exposure [30,71].

## 4. Smart Materials in Orthodontics

### 4.1. Shape-Memory Polymers

Shape-memory polymers are an emerging class of polymers with various biomedical and dental applications. These polymers are dual-shape materials belonging to the group of “actively moving” polymers that can change their shapes, a temporary shape being obtained by mechanical deformation and a permanent shape being obtained from the subsequent fixation of that deformation [72]. The changes in these shapes of polymers can be induced by external stimuli such as heat, light, infrared radiation, electrical and magnetic fields, and immersion in water [34]. The application of external stimuli to these polymers then introduces a departure from the as-received shape to a reversible new configuration.

**Figure 3:**
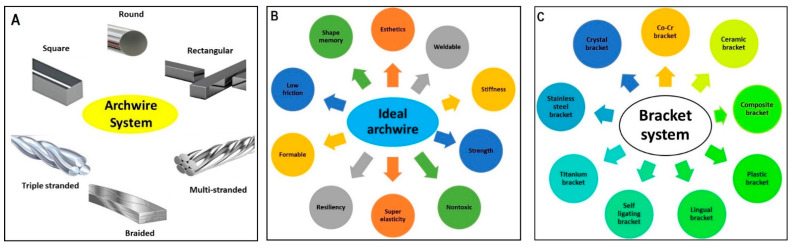
(**A**) Different archwire system being used for orthodontic treatment, (**B**) properties of the ideal archwire system, (**C**) different bracket system being applied for orthodontic treatment.

In orthodontics, shape-memory polymers have a high potential for functions and esthetic appearances. These materials are valuable in manufacturing polymeric transparent wires with minimum stiffness, which could then be transformed into arch wires with a predetermined elastic modulus upon exposure to a stimulus such as light or heat [34]. Shape-memory polyurethane (P.U.) with poly (ε-caprolactone) (PCL) was prepared to produce P.U. wire. Shape-memory PU consists of hard and soft segments; the hard segments play the role of physical crosslinks with a high melting temperature via hydrogen bonding and crystallization [73]. The soft segments provide the reversible phase transformation necessary for achieving a shape memory effect. Combining these two features improves the flexibility and mechanical resistance of P.U. wire in order to correct misaligned teeth through thermal heating from body temperature (Figure 2) [73,74]. Therefore, shape-memory P.U. wires have a great potential for orthodontic applications and provide a satisfactory esthetic appearance. However, a detailed investigation on the PCL counterpart is needed, as it could undergo rapid hydrolytic degradation depending on the pH of the environment [75]. The archwire system Figure 3A and archwire system with ideal properties has been presented for the treatment of orthodontic.

### 4.2. Self-Healing Materials

**Figure 4:**
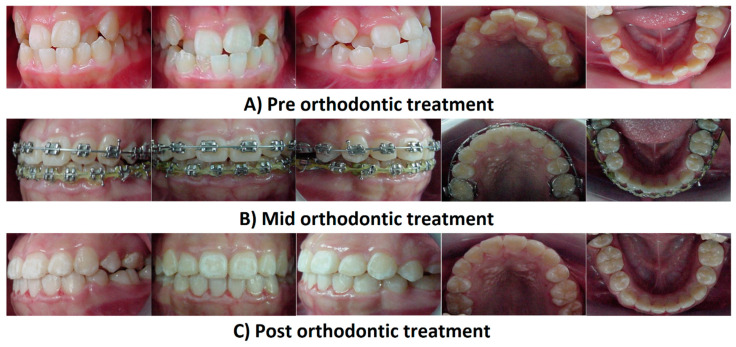
Photographs of the fixed appliance for orthodontic treatment using shape-memory P.U. wire. (**A**) Pre-orthodontic treatment; (**B**) Mid-orthodontic treatment; and (**C**) Post-orthodontic treatment.

**Figure 5:**
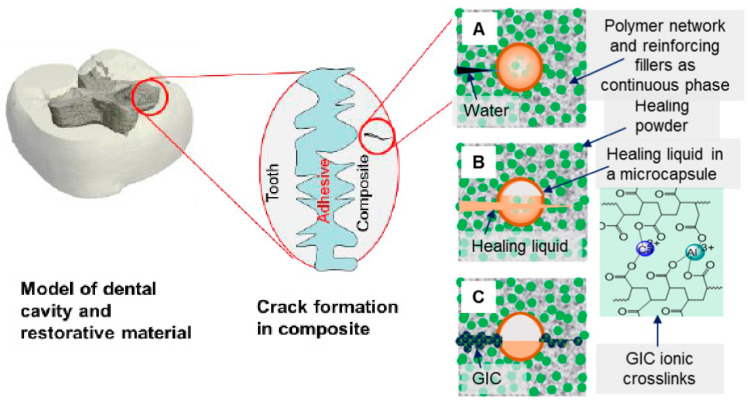
Self-healing steps of SHDC. (**A**) Fracture: A crack forms and water comes in, (**B**) Delivery: a microcapsule is broken due to crack propagation and releases HL, (**C**) Healing: the HL reacts with HP and produces GIC with the ionic crosslinking network and reprinted with permission from published work of Huyang, G. et al and coworkers [77].

Over the past decade, there has been intensive research on designing and manufacturing smart and self-healing synthetic systems that can mimic the behavior of natural biological systems and efficiently repair damage. Different bracket system has been used in orthodontic treatment as mentioned in in Figure 3C. Efforts are being made to enhance the self-healing properties of polymers and their composites [73,76]. When a crack initiates, the HL is released from microcapsules and reacts with HP to form a reparative GIC that fills and seals the fissure in Figure 4. Huyang et al. reported a new model of self-healing dental composites (SHDC) as presented in Figure 5, and White et al. have developed a dental composite by combining the fracture-release-heal process in the Urbana Champaign (UIUC) self-healing model with glass ionomer cement (GIC) [77,78]. Conventional dental composites commonly have two key components:

(1) Dimethacrylate-based polymers to provide a resin network.
(2) Reinforcing filler particles treated with coupling agents to bond the resin to the particles.

The SHDC has two additional components over the conventional dental composites:

(1) Healing powder (HP): strontium fluoroaluminosilicate particles.
(2) Healing liquid (HL): aqueous polyacrylic acid solutions encapsulated in silica microcapsules to prevent premature release during composite preparation.

When a crack initiates, the HL is released from microcapsules and reacts with HP to form a reparative GIC that fills and seals the fissure in Figure 4.

Experimental data showed satisfactory values for the elastic modulus of SHDC. The healing process was confirmed by mechanical, morphological, and chemical studies. Besides the self-healing characteristics, the combination of antibacterial and remineralizing capabilities is another breakthrough in the development of SHDC. In other studies, Dimethylaminohexadecyl methacrylate (DMAHDM) was added as an antibacterial agent, while the presence of nano-calcium phosphate governed the remineralization process. DMAHDM reduced the biofilm formation fourfold compared to that of the control, suggesting its antibacterial efficacy. The coexistence of a self-healing microcapsule increased up to 7.5%, and DMAHDM and nano-calcium phosphate did not obstruct the self-healing mechanism, signaling its relevancy for a broader range of applications [79]. An almost similar study confirmed that the addition of a 7.5% microcapsule resulted in the formation of an excellent self-healing composite with superior mechanical properties even after being subjected to water aging for six months, indicating its durability [79]. Apart from that, the introduction of a silane coupling agent in SHDC was reported to produce a composite with a significantly higher resistance to crack propagation [80]. Another advantage of SHDC is that the materials used in this system are clinically tested and biocompatible, and can be readily used for clinical applications. The orthodontic application of this innovation may also involve polymer brackets and arch wires. The integration in these materials of nanosized bubbles filled with auto-polymerized monomer may result in fewer bracket and wire breakages. The fracture of the bracket or wire would cause bursting of the nanosized bubbles and exposure of the monomer to the air, thus promoting the polymerization and filling of the crack-induced gaps [34].

### 4.3. Self-Cleaning Materials

The issue of plaque retention on brackets and microbial attachments onto biofilms has raised major concerns in dental material research. It is fascinating to develop a material that could clean itself of mainly organic and inorganic precipitations [81,82]. Earlier research in this area adopted the concept of the “lotus effect” inspired by microscopic bumps on a lotus leaf that transform its waxy surface into an extremely water repellent or superhydrophobic substance. Apart from the “lotus effect”, some synthetic self-cleaning materials use the opposite property of “super hydrophobicity” or catalytic chemical reactions. The development of self-cleaning surfaces would positively impact dentistry, such as increasing the longevity of denture base materials and implants through the protection against bacteria and the inhibition of biofilm formation [34,83].

A newly developed coating material with self-cleaning properties has been introduced to inhibit biofilm formation. Fluoroalkylated acrylic acid oligomer (FAAO) was incorporated with a photo-activated acrylate material at various concentrations [84]. Biofilm formation on the surface was assessed using *Streptococcus mutans* biofilms inside an oral simulator in vitro. The results demonstrated an increase in the concentration of FAAO in the coating materials, and an enhanced surface hydrophilicity and oil-repellency. Assay studies indicated that the amount of biofilm retained on the coating materials gradually decreased when the concentration of FAAO increased in the materials. Therefore, it is observed that FAAO-incorporated materials possess self-cleaning properties and effectively prevent biofilm formation on the surface. Hydrophilic materials tend to form a thin water layer on their surfaces, which hinders protein binding [85]. The natural repulsive forces due to the different hydrophobic cell envelopes and hydrophilic material surfaces obstruct the adhesion mechanism.

### 4.4. Biomimetic Adhesives

The term biomimetic is derived from the Greek word “bio”, meaning living, and “mimetic”, meaning imitating or resembling. The biomimetic concept refers to how creatures ingeniously use natural elements to solve environmental problems [34]. Gecko footpads and mussel adhesion mechanisms are two remarkable natural examples of bonding that have been well studied so far [86,87]. Geckos are lizards that exhibit the mechanical principle of “contact splitting,” where they can attach to a surface with a strong but temporary adhesion. The gecko’s foot has a flat pad that is densely packed with fine hairs split at the ends, resulting in an increased number of contact points compared to if the hairs were not split. Several contact points between these hairs and a surface lead to a significant increase in the adhesion force through localized van der Waals forces [87]. Although this binding mode seems ideal, it is only suitable in dry environments, not on wet surfaces. Then, another natural example of bonding is mussels overcoming the weakness of the gecko mechanism. Combining the gecko and mussel adhesion mechanisms leads to a new adhesive material called “geckel”, which functions like a sticky note and exhibits a strong yet reversible adhesion in both air and water [86]. Mussel-mimetic polymers have an amino acid called *L*-3,4-dihydroxyphenylalanine (DOPA), found in high concentrations in the “glue” proteins of mussels [88,89].

Contrary to the gecko-based approach, pillar arrays (400–600 nm in diameter and length) coated with the mussel-mimetic polymer improved wet adhesion 15-fold more than uncoated pillar arrays [88,90,91]. A deep understanding of this type of biomimetic adhesive could be useful in manufacturing brackets. Brackets having bases with pads mimicking the gecko foot and covered with a layer of DOPA would provide a sufficient bond strength to sound enamel without prior enamel conditioning and would avoid color and structural alterations of enamel [92,93].

## 5. Conclusions

Recent developments of polymeric orthodontic materials have the potential to overcome the past materials’ weaknesses, with improved mechanical properties and, most importantly, BPA-free structures. Furthermore, the emerging class of polymers with fascinating properties, such as the dual functions of shape-memory polymers, self-healing, self-cleaning, and biomimetic adhesion, would improve the effectiveness and longevity of orthodontic treatments. A deep understanding of the structure and intermolecular interactions involved within base monomers is fundamental to synthesizing and developing new materials. Nevertheless, the newly synthesized materials’ safety regulations and cost-effectiveness need to be addressed before they are introduced into the market. Polymer science engineers, chemists, and orthodontists should be working together to accelerate the innovation and technologies involved in manufacturing future polymers with superior properties and biocompatibility.

## Data Availability

The data presented in this study are available on request from the corresponding author.
